# Active Play as a Window Into the Worlds of Twins and Triplets With Autism

**DOI:** 10.1155/oti/4415279

**Published:** 2025-03-10

**Authors:** Marie Abu Itham, Serene Kerpan, Robert Balogh, Meghann Lloyd

**Affiliations:** ^1^Faculty of Health Sciences, Ontario Tech University, Oshawa, Ontario, Canada; ^2^Faculty of Education, Vancouver Island University, Nanaimo, British Columbia, Canada

**Keywords:** autism spectrum disorder, communication, multiples, parents, physical play

## Abstract

**Background:** Research indicates that play can facilitate communication and emotional connection for children with autism and their parents. Currently, there is no research exploring the perceptions that parents of twins and triplets with autism have regarding their children's play, despite these parents' unique opportunity to observe and interpret the play behaviours of multiple same-age, same-diagnosis children raised in the same home environment. The purpose of this descriptive phenomenological study was to describe the value that parents of twins and triplets with autism place on active play.

**Method:** The researchers used purposive sampling to recruit abroad sample of mothers (*N* = 9) of twins and triplets with autism aged 4–11 years old. Participants took part in one semistructured online interview with the researcher which was then thematically analyzed.

**Results:** One central theme emerged from our analysis. This theme is entitled active play as a “window into a child's world” and contains five subthemes: (1) parent perceiving child's strengths and weaknesses in active play, (2) parent facilitating active play experiences, (3) parent perceiving child's intrinsic motivations for active play, (4) parent interpreting child's active play behaviours, and (5) active play experiences as a medium for parent/child communication.

**Conclusions:** These findings suggest that parents value active play because it affords them opportunity to observe their children's characteristics through their active play behaviours (e.g., strengths and weaknesses) and use what they have learned to facilitate new active play experiences that encourage overall development. Through active play, parents also practice communicating verbally and nonverbally with their child.

## 1. Introduction

Autism spectrum disorder (henceforth referred to as autism) is a neurodevelopmental disorder characterized by differences in social communication and restricted, repetitive behavioural development impacting the way an individual communicates and interacts within their environment [1]. Global prevalence data estimates that one in 100 children has a diagnosis of autism [2] and studies exploring rates of autism in sets of twins demonstrate that the probability of receiving a diagnosis increases to 88% if an identical twin sibling has already received a diagnosis and 31% if the same is true for a nonidentical twin [3]. Even within twins, the social communication and behavioural differences exhibited by children with autism exist on a spectrum and so there is substantial variability in their expression [4].

The social communication and behavioural differences that children with autism experience can be seen in the way they relate to others and engage in daily occupations such as play [1]. For example, children with autism often struggle to pick up on emotional and interpersonal cues [5] and may employ fewer verbal and nonverbal communication methods during social interactions [6]. These differences can also impact the way that they engage in play by themselves and with others [7]. For instance, children with autism often show fewer expressions of fun and spontaneity in their play [8], despite these characteristics being considered hallmarks of play in children who are neurotypical [9].

Previous research has demonstrated that parents of children with autism are able to gain perspective into the inner experiences of their children through active play, specifically their children's perspectives, interests, and strengths [10, 11]. Active play refers to any type of freely chosen and unstructured play where a child is using total body movements to exert their energy in a way that they are choosing because it is fun [12]. In this active play context, parents of children with autism have discussed that they take note of their children's differences in play, while simultaneously celebrating their capabilities [10, 13]. In both observing and participating in play with their children with autism, parents are able to gain insight into their children's inner worlds [13].

In addition to providing insight into their child's world, play can also serve as a platform for parents to communicate with their children with autism [11, 14]. Research indicates that parents of children with autism may experience a higher quality of verbal and nonverbal communication with their child when they are open to their child's point of view and seek to better understand their perspective [11]. However, while parents often seek emotional connection with their children by matching their child's smile or laugh at a particular object or movement, research indicates that children with autism may spend more time engaging with objects than with their parent during parent–child play experiences [15]. These findings suggest that while parents may be sensitive to their children's emotions during play [14], they may have difficulty sustaining this connection in their children's inner worlds [15].

Previous research exploring the relationships between parents of triplets, twins, and singletons who are neurotypical indicates that parent–child communication may be more difficult in the context of multiples, given the extra demand on parents [16]. Specifically, parents of triplets may be less likely to be sensitive to their children's emotions, preferences, and needs during play [16]. Considering the communication differences that children with autism display [11, 15], it is possible that parents of twins and triplets with autism may face barriers in connecting with their children's world. Verbal and nonverbal communication between parent and child is essential for children with autism as it provides an opportunity for children to understand and practice social interactions from an early age [17]. Although there is some research indicating that parents of triplets who are neurotypical may face barriers in building strong emotionally connected relationships with their children due to the increased demand on their attention [16], there is an overall paucity of research on the topic. Furthermore, there is no research exploring the ways that parents of twins and triplets connect with their children through active play.

The purpose of this descriptive phenomenological study was to explore and describe the perceptions that parents of twins and triplets with autism have regarding their children's active play behaviours. This study was guided by the following question: What value do parents place on active play for their twins and triplets with autism?

The choice to use person-first language (i.e., child with autism) in this study rather than identity first language (i.e., autistic child) was informed by parents' report of their own preferences [10].

## 2. Materials and Methods

### 2.1. Study Design

A descriptive phenomenological approach was chosen to guide the research process [18] because this methodology employs both the researcher and participants with lived experience of the phenomenon, in a dynamic and inductive process of developing an accurate picture of the phenomenon in order to generate new knowledge [18]. The researcher used the consolidated criteria for reporting qualitative research as the framework to guide reporting of methods and data [19].

### 2.2. Positionality and Bracketing

Throughout the research process, the researcher took on a phenomenological mindset, engaging in a variety of self-reflection strategies, including positionality and bracketing, to reduce the effect of any preconceived notions or prior experience [20]. The researcher approached the research process from a social constructivist framework, a perspective that influenced her decision to gather descriptions of the phenomenon by asking individuals about their own views of their experiences. In line with this approach, a bracketing exercise was used to facilitate the setting aside of her own experiences from the descriptions and perceptions that the participants expressed [21]. A segment of this bracketing exercise is included below:

I am not a twin or triplet nor do I have autism or a loved one with autism. During my undergraduate degree in kinesiology and now as a masters student working in the realm of adapted physical activity, I've had multiple opportunities to work with children with autism in a physical activity setting and I've found myself appreciating how each one is so unique. I've also begun to appreciate interactions with parents, observing how they communicate and interact with their children. Overall, I've found that these experiences have cultivated curiosity in me, helping expand my lens outside my own life experience.

### 2.3. Participants

This study was approved by the Research Ethics Board of the author's academic institution. Eligible participants were parents or legal guardians of 4–11-year-old multiples (e.g., twins or triplets) with autism; each child in the multiple set needed to have a diagnosis of autism, as reported by the parent/guardian. Other eligibility criteria included the ability to speak and read English and access Google Meet video-conferencing [10]. This age band was chosen due to the developmental window associated with active play behaviours and because most children with ASD are diagnosed by this age range.

Nine participants were recruited using purposive sampling techniques, included in this recruitment technique was contacting parents who had provided consent to be informed if they met the eligibility criteria for future studies (e.g., twins/triplets with ASD between the ages of 4 and 11 years), and the recruitment materials were shared with a support group specifically for parents of multiples with ASD This sample size was sufficient to reach thematic saturation [22] and was within the recommended range for phenomenological studies [21]. All participants filled out a digital consent and demographic form and were given a $50 Amazon gift card upon completion of a one-on-one virtual interview [10]. The sample recruited for this study included eight mothers of twins with autism and one mother of triplets with autism (*N* = 9); four from Canada, four from the United States, and one from Portugal. In total, the results of this study pertain to 19 children (11 females, 8 males) between 5 and 11 years of age (Table 1), some of whom communicated primarily verbally and others primarily nonverbally.

### 2.4. Procedure

The semistructured interview guide (Table 2) was pilot tested with a mother of 4-year-old twins who are neurotypical. Before beginning each interview, the researcher laid out an operational definition of active play to ensure that each participant understood the scope of the topic. Specifically, parents were told that active play is any kind of unstructured play where children use their whole body in their play; when they are playing actively, they are using their energy in a way that is fun and towards something they are choosing to do [12]. The primary researcher conducted all nine 60–90-min virtual interviews in November 2021; Google Meet was used for each interview and Otter.ai was employed to audio-record, transcribe, and later deidentify transcripts [10]. Pseudonyms were used in place of all child names in the transcripts.

The researcher wrote reflexive journal entries immediately following each interview and throughout the research process to record contextual information (e.g., participant's emotions) and personal reflections [23]. The researcher also recorded an audit trail, keeping detailed notes of each step of the research journey [20].

### 2.5. Data Analysis

The researcher sent participants a verbatim transcript of their interview 4–5 days following their interview, providing each participant an opportunity to request changes they would like made (i.e., additions, clarifications, and omissions). Five of the nine participants responded, and only two requested minor changes. The researcher then reviewed each transcript multiples times over the course of a month to familiarize themselves with the overall narrative and recorded notable phrases or ideas for the purpose of identifying key themes [24]. Each participant was sent an individualized synthesized member checking document with preliminary themes, supporting quotes, and a list of questions to help guide them through the process of reflecting on their document (e.g., “Do the findings capture the essence of what you shared?”). Participants were once again given the opportunity to respond with any revisions they wished to make. Three out of the nine participants responded, each expressing satisfaction with the preliminary findings [10].

Following this member checking process, the researcher analyzed the data through a combination of deductive and inductive content analysis. NVivo was used to support analysis, and the researcher utilized the deductive content analysis framework suggested by Bradley et al. [25] to develop the initial coding structure. Initial conceptual codes were developed further using some themes identified through researcher memos and categories of play identified in Coopers' Conceptual Model of Children's Play [26]. At this point, the initial coding structure was in place and the researcher began to engage in an iterative coding process wherein codes and subcodes were created, split into more specific codes, and/or renamed to better reflect concepts and themes as they developed over time. This process was conducted by the primary researcher, with support from her supervisor as they met regularly to discuss the developing codebook.

## 3. Results

During interviews, parents expressed that they value their children's active play behaviours for how they serve as a window into each child's world. Parents' discussions of this “window” can be categorized into five main themes: (1) parent perceiving child's strengths and weaknesses in active play, (2) parent facilitating active play experiences, (3) parent perceiving child's intrinsic motivations for active play, (4) parent interpreting child's active play behaviours, and (5) active play experiences as a medium for parent/child communication.

### 3.1. Parent Perceiving Child's Strengths and Weaknesses in Active Play

During interviews, parents described how observing their children in active play gave them a front row seat to their children's strengths and weaknesses in a variety of areas including motor skills, problem solving, and creativity in coming up with play ideas. In some cases, parents reflected on the talents they observed in their children.

…my daughter actually struggles more with like the sportsmanship kind of stuff and you know, like losing a game…Whereas my son … actually got very good at that. (P7)

I always think like, if only they could be persuaded or taught to like, follow instructions, they would be amazing athletes, because they are just natural strength and like athletic skill is like, amazing to me. (P6)

### 3.2. Parents Facilitating Active Play Experiences

Parents described how observing their children's behaviour in active play enabled them to engage with their child and their play both directly and indirectly. For example, parents discussed modelling certain play behaviours during parent-child play to help their child notice that a particular play option was enjoyable and setting up particular activities in play environments based on what they had previously observed their children enjoying.

…let us do something fun. Go for a bike ride…Everyone likes a bike ride… I'll make goals like, you know, Dominic, go first. And Dominic's gonna stop at the green box. Or you are gonna ride your bike to the red car. (P2)

…so, when they would come to me, they'd be like, I do not know what to do…And I'd say okay, well, you know, here's this, all these little invitations to play. Why do not you go pick one? And then after about six months of me just reiterating all the different things that they could do to play, at a certain point, especially for Gabby, I saw this big switch where then she understood all of the options that were there and different combinations that she could do and now when she goes outside, she just does them. (P4)

### 3.3. Parent Perceiving Child's Intrinsic Motivations for Active Play

Parents discussed their perceptions of each of their children's intrinsic motivations for play, often citing their children's behaviour, personality, commonly chosen play activities, and level of enjoyment. Parents gave examples such as movement, people, mischief, competition, performance, textures, music, curiosity, and creativity.

She likes to move her body and she likes to move it often. She enjoys swinging, bouncing, hiking, um, things like that. (P5)

…if he if he sees anybody, he's right over there saying hi… I think he wants to be accepted. Like he just, he wants people to know he's there and invite him over and include him. (P1)

### 3.4. Parent Interpreting Child's Active Play Behaviours

Parents often reflected on their interpretations of the active play behaviours they observed their children engaging in. Some parents talked about their children's play as being typical for children their age, while others mentioned that they saw more conventional play behaviours when their child was younger or more so as they got older. Parents also reflected on their children's play behaviours changing as they learned new play skills, sometimes attributing these changes to a child's expanding play skill repertoire as they had new experiences and other times attributing them to the opportunity their children had for frequent play experiences with their twin or triplet. Finally, parents also discussed the impact of facilitators (e.g., being outside in the yard) and barriers (e.g., preference for iPad games) on their children's active play behaviours.

One's running the whole dialogue. But she'll prompt the other one what to say, and then she'll say her response … “and then you say, “Oh, wow”. And then I say, “yeah” … I've never seen anyone else do that. (P3)

…And practice on each other, so when you go out into the real world, like, you know, so when he goes to school, when Henry goes to school to play with his friends, he's kind of practiced playing with Victoria, like taking turns and sharing so that it generalizes into, you know, the school environment or the park environment. (P1)

### 3.5. Active Play Experiences as a Medium for Parent/Child Communication

During interviews, parents discussed a variety of ways, both verbal and nonverbal, that their child used to communicate with them (i.e., child to parent communication). For example, parents frequently described that observing their children's behaviours, emotions, and sensory regulation during active play helped them to perceive their children's enjoyment of activities and/or dislike of an activity or environment.

… you can kind of see her build up when she's about to get into an unpredictable, potentially stressful to her situation … I do not know if anybody else could, but I could watch her trying to dial it down... I could see her not even resorting to that stim and just trying to kind of ease into it… sometimes it worked…other times it did not. You know, she would give it she would give it her level best. (P5)

…he liked his friends in the pool, but … they were not letting him do his like usual stuff, or in his way or whatever, you know? … he was able to like do his usual thing that he liked. (P9)

## 4. Discussion

The purpose of this descriptive phenomenological study was to gather descriptions from parents of twins and triplets with autism about the value they place on play for their twins and triplets with autism.

### 4.1. Parent Perceiving Child's Strengths and Weaknesses in Active Play

Research shows that parents of singletons with autism value play because it provides an opportunity to observe their children's strengths and weaknesses [13]. Parents in our study talked about placing value on their children's active play because of the opportunity it afforded them to be able to distinguish between each of their twins' and triplets' unique abilities and challenges. This is particularly relevant considering previous research indicating that parent of triplets who are neurotypical may be less attuned to each of their children because of the extra pressure of raising a multiple set of three children (e.g., higher rates of stress and maternal depression) [27]. Adding to this dynamic, research shows that raising one child with autism can put a strain on parents [28]; while there is limited research exploring the relationships between parents and their twins and triplets with autism, it is conceivable that this strain might be amplified if one were raising multiple children with autism. However, the findings of our study are similar to those of Burgoine and Wing [29] whose case study of triplets with Asperger's syndrome highlighted the mother's capacity to be emotionally connected with all three of her children and documented a variety of ways that she was closely involved in her children's lives. It is possible that the level of attunement that parents in our study showed to their children's unique strengths and weaknesses is amplified due to the fact that parents were explicitly discussing their children's play. Considering these parents volunteered to participate in this study, it is also possible that they are more engaged in their children's play than other parents might be and therefore have more to say on the topic. Even still, it may be possible that active play provides a helpful context for parents to observe their kid's strengths and weaknesses in flexible home and community environments in a way they do not get to see in other structured contexts (e.g., academics or therapy). In light of these findings, there is a need for more research exploring how parents of children with autism may gain valuable insight into their children's strengths and weaknesses by observing them engaging in free play.

### 4.2. Parents Facilitating Active Play Experiences

Parents talked about encouraging their children to pursue active play experiences based on their preferences. In some cases, parents discussed setting up play spaces structured with specific play stations that they had previously observed their children enjoying (e.g., slip-n-slide, swing set, and sandbox). Beyond active free play, parents also talked about using their observations of their children's strengths to inform their decisions regarding what recreational programs to sign their kids up for, such as cheerleading or rock climbing. These findings regarding a parent's attunement to their children's play experiences are corroborated in a study conducted by Di Renzo et al. [11] who found that parents of singletons with autism focus on their relationship with their children during play, rather than on the activity itself, and as a result are better able to perceive their children's desires and challenges in play and respond accordingly [11]. This same dynamic was present in the current study with parents of twins and triplets with autism as parents encouraged and challenged their children based on their observations of each child's interests in play. These findings indicate that parents of twins and triplets with autism play an important role in encouraging their children to engage freely in active play by embracing each of their children's diverse play behaviours.

Similarly, parents can also play an important role in addressing the unique challenges their children exhibit in active play [11]. This dynamic was described by parents in the current study as they demonstrated an ability to understand the task and environmental constraints that impact play for their twins and triplets. Although the concept of a child freely choosing to play an activity is integral to the definition of active play, parents in the current study discussed that their children did not always know how to make such decisions; those who were younger or who had a more significant challenges related to their autism sometimes needed guidance from parents about what they could play with. In response to these difficulties, parents took varying approaches to facilitate positive play experiences for their children based on each individual child's unique abilities and needs. Previous literature supports these findings by suggesting that parents can help their children become more physically active by assisting them in coming to an understanding of what types of active play they enjoy [10, 30]. Individualized, strength-based intervention strategies such as this where children are encouraged to pursue their interests are appropriate as they can take place in a child's natural environment [31].

Our findings indicate that parents value the opportunity that active play affords them to better understand each of their children and to facilitate positive play experiences for their children. However, this remains a difficult task due to the diverse social, behavioural, and motor differences of children with autism; and while parents are experts in their own children, most do not have education in early childhood education or physical education training and as a result do not always have all the information needed to facilitate positive active play experiences for their children [31]. For this reason, parents may benefit from coaching support from community organizations, providing them with creative ideas for play activities to try at home as well as opportunities for community play [31]. In this way, parents will feel more supported, motivated, and encouraged to facilitate individualized physical activity opportunities for their children [31]. Further research is needed to understand the needs that parents have in facilitating active play for their children and how community organizations may be able to come alongside them through parent-mediated active play interventions for children with autism.

### 4.3. Parent Perceiving Child's Intrinsic Motivations for Active Play

Research indicates that children with autism may be intrinsically motivated to engage in their favourite activities for similar reasons to those of children who are neurotypical (e.g., feelings of enjoyment and accomplishment) [10, 32]. These findings are particularly illuminating in light of previous research suggesting that children with autism have strong preferences for certain activities simply because such activities help to ease anxiety or other negative emotions [33]. The parents in our study discussed a variety of reasons why their children found play to be valuable and enjoyable, giving examples such as children running around the playground to explore and satisfy their curiosity or making creations out of sticks and mud in the backyard out of a desire to be creative. These findings are in line with previous research stating that, despite all their similarities, same-sex twin pairs can be differently motivated to engage in physically active leisure based on their preferences and what they hope to get out of the activity [34]. It is possible that these findings are amplified by the fact that this study is pertaining to twins and triplets and that this theme of intrinsic motivations may not have come through so strongly were it not for parents comparing and contrasting their perceptions of twins and triplets differing play choices despite being in the same environment. Nevertheless, the intrinsic motivations of children with autism are important to consider as researchers have found that singletons with autism show marked improvements in joint attention, language, social communication, emotional regulation, motor skills, self-confidence, and positive self-image when they are allowed to engage in play that they are particularly interested in [35].

Research exploring play interactions between parents and singletons with autism has revealed similar findings as parents have expressed that they are better able to connect with their child when they understand why they are enjoying a particular play activity, even if that activity is repetitive or otherwise nonnormative [13, 36]. For example, some parents of singletons have expressed that parent–child play interactions are more rewarding and enjoyable when the parent engages in the play in a way that aligns with what the child is hoping to get out of it, rather than trying to assert their own wishes onto the play [36]. Parents in our study expressed similar sentiments of enjoyment and satisfaction when observing their children engaging in play that brought them joy. These findings are particularly meaningful as there is a paucity of research exploring how the innate interests of children with autism may serve to facilitate positive play experiences [35]. It is possible that parental attunement towards children's intrinsic motivation for play, may serve to strengthen relational bonds between parents and twins and triplets with autism.

### 4.4. Parent Interpreting Child's Active Play Behaviours

One unique aspect of the current study is that parents had the opportunity to talk about their twins' and triplets' play as it evolved throughout their childhood, to date. As a result, the findings captured a variety of parents' impressions of their children's play over time. In particular, parents reflected on instances when their children played in ways that they perceived as being typical for childhood play (e.g., a game of tag) as well as giving examples of their children exhibiting what they perceived to be unique or unexpected behaviours (e.g., repeatedly running along a fence line). These findings reflect similar themes to those found in a study conducted by Mitchell and Lashewicz [13] who identified that the parents of singletons with autism in their study each went through similar processes coming to terms with their children's unique or “quirky” play behaviours, ultimately coming to see such play behaviours in a positive light. Similarly, while the parents in our study described some of their children's play as being different from what they might expect to see in children who are neurotypical, they often framed such play behaviours as intriguing rather than concerning because of the way it gave them a window into their child's inner world. While there is much research framing the play differences of singletons with autism as abnormal [37, 38], these findings suggest that parents of children with autism may view their children's play as more complex than simply being “deficient or typical.” Moreover, the findings of the present study suggest that parents of children with autism may enjoy and encourage some of their children's play behaviours that might generally be seen as atypical because they value the opportunity this type of play provides them to connect with their children.

### 4.5. Active Play Experiences as a Medium for Parent/Child Communication

Play experiences between parents and children with autism have been shown to provide an effective opportunity for the development of quality communication between parent and child [11, 39]. Research indicates that parents of singletons with autism report high stress levels in part due to the reality of their child's lower communication skills and presence of socially disruptive behaviours [28], but that parent–child play experiences can have a positive effect on this communication [39]. Similarly, the findings of the current study also suggest that active play experiences may have a positive modifying effect on the quality of relationship between parents and twins and triplets with autism. Parents in the current study discussed that they valued opportunities to observe their children in active play because they could come to better understand their children's preferences; they were able to connect with their children on a deeper level as they observed the types of play that brought their children joy and those that induced anxiety or fear. For example, one mother described her twin boys with autism as being “silly,” which she later attributed in part to their tendency to laugh and sing while running, climbing, and jumping. It is important to highlight that similar to Solomon et al.'s [39] findings, both parents of children who are verbal and those who are primarily or completely nonverbal expressed an increased ability to connect with their children through active play. It is possible that in active play, parents are able to be more attuned to their children's nonverbal communication styles. These findings point to the value that parents place on active play as a window into their child's world.

Previous research has indicated that parents of singletons with autism have an ability to be sensitive to their children's positive and negative responses to sensory stimuli, for example, a child's enjoyment of watching and chasing bubbles as compared to a dislike of the feeling of being tickled [14]. Parents in the current study demonstrated a similar ability to not only perceive their children's positive and negative responses to sensory stimuli but to also understand what their child might be communicating through a particular response to such stimuli. These findings are further emphasized by the fact that parents demonstrated the ability to perceive and respond to the sensory experiences of both/all their children with autism even though these were different from child to child. These findings point to the value that parents of children with autism may place on sensory experiences as opportunities to connect with their children's experiences. While much autism research paints sensory play as detracting from the overall play experiences of children with autism [40–42], these findings point to the important role they may play in facilitating close parent–child relationships among parents and children with autism.

## 5. Future Research

There is a need for further research exploring the positive effects that active play can have on the quality of verbal and nonverbal communication between parents and children with autism. It is possible that this phenomenon may be more prominent in relationships between parents and multiples with autism as these parents may spend more time engaged with their children's autistic worldviews as a function of having two or three children with autism. Conversely, it is also possible that the relationship between active play and parent–child communication may be stronger between singletons with autism and their parents; where parents of multiples must divide their attention between two or more children, parents of singletons can focus on just one child. This area of research is particularly meaningful as previous research has identified a positive correlation between relationship quality, warmth, and verbal praise for parents of children with autism as well as fewer internalizing and antisocial behaviours among children with autism [43]. With this in mind, there is a need for future research to further explore the positive effect that active play experiences can have on parent–child communication among families with children with autism.

## 6. Strengths and Limitations

One strength of the study was the international sample [20] which enabled the researchers to explore which features and values were specific to a participant's context and which were common to parents of twins and triplets [44]. The inclusion of parents of both twins and triplets in the study could be viewed as a limitation due to the different life experiences of twin and triplet parents. However, because this is the first study to explore this phenomenon, the researchers decided to include them in order to gather as much data as possible. Furthermore, the researchers accepted interviewees on a first-come, first-serve basis and therefore, the presence of self-selection bias is possible [45]. To draw a wider sample and mitigate this bias, the researchers offered a $50 Amazon gift card.

## 7. Conclusions

The findings of this study highlight how perceptive parents of twins and triplets with autism are regarding their children's active play. During interviews, parents described the value they placed on active play as an opportunity to better understand the strengths, weaknesses, and intrinsic motivations of each of their children. These findings indicate that active play is valuable for parents because it serves as a helpful context for parents and children with autism to practice communicating with one another, both verbally and nonverbally. This connection may be especially important for parents of children with autism as this population often reports higher levels of stress due to the realities of parenting a child with social communication and behavioural differences. Parents in this study also discussed that they valued their children's active play because they are able to use what they have observed of their children in play to facilitate further development of play skills. These findings are meaningful given research proposing strength-based models as beneficial intervention strategies for improving the overall quality of life and wellbeing of children with autism. Future research should explore how parents and community recreation providers can work together to develop strength-based interventions for children with autism, where children can learn new skills while being encouraged and given opportunities to use their strengths in play.

## Figures and Tables

**Table 1:** Demographic information of children as reported by participants.

**Participant**	**Age**	**Zygosity**	**Sex**
1	6	Fraternal	Male, female
2	7	Two identical, one fraternal	Male, male, male
3	5	Fraternal	Female, female
4	10	Identical	Female, female
5	9	Fraternal	Female, female
6	7	Identical	Male, male
7	11	Fraternal	Male, female
8	10	Identical	Female, female
9	7	Fraternal	Male, female

**Table 2:** Semistructured interview guide.

**Question**
1. Can you tell me about your family?
2. Can you tell me about your twin's/triplet's play when they were 1–2 years old? 3–5 years old? School age?
3. Can you tell me about how your twins/triplets play together?
4. Think back to the most recent time that you noticed that your twins/triplets were playing really well together. What stuck out to you?
5. In the course of a week, what kind of play activities do you do with your twins/triplets?
6. If you had some friends or family over for a visit in the backyard, would your twins/triplets play with the other children?
7. Can you tell me about a time when your child was playing, and you felt proud of them?
8. If you could do anything on a Saturday afternoon with your children, what would you do?

## Data Availability

This is a qualitative study; the transcripts are not publicly available.
